# The Omega-3 Fatty Acid Docosahexaenoic Acid Attenuates Organic Dust-Induced Airway Inflammation

**DOI:** 10.3390/nu6125434

**Published:** 2014-11-27

**Authors:** Tara M. Nordgren, Taylor D. Friemel, Art J. Heires, Jill A. Poole, Todd A. Wyatt, Debra J. Romberger

**Affiliations:** 1 Pulmonary, Critical Care, Sleep and Allergy Division, University of Nebraska Medical Center, Omaha, NE 68198, USA; E-Mails: tnordgren@unmc.edu (T.M.N.); taylor.friemel@gmail.com (T.D.F.); aheires@unmc.edu (A.J.H.); japoole@unmc.edu (J.A.P.); twyatt@unmc.edu (T.A.W.); 2 VA Nebraska-Western Iowa Health Care System, Omaha, NE 68105, USA; 3 Department of Environmental, Agricultural, and Occupational Health, University of Nebraska Medical Center, Omaha, NE 68198, USA

**Keywords:** docosahexaenoic acid (DHA), organic dust, airway inflammation, specialized pro-resolving mediators (SPM)

## Abstract

Workers exposed to organic dusts from concentrated animal feeding operations (CAFOs) are at risk for developing airway inflammatory diseases. Available preventative and therapeutic measures for alleviating dust-induced lung disease are inadequate. Because omega-3 fatty acids can mitigate inflammatory processes, we aimed to determine whether nutritional supplementation with the omega-3 fatty acid docosahexaenoic acid (DHA) could reduce the airway inflammatory consequences of exposures to organic dust. Aqueous extracts of organic dusts from swine CAFOs (ODE) were utilized. In DHA-pretreated human bronchial epithelial cells, lung fibroblasts, monocyte cell cultures, and precision-cut murine lung slices, we found that DHA pretreatment dose-dependently decreased ODE-induced inflammatory cytokine production. To determine the *in vivo* significance of DHA, C57BL/6 mice were orally administered DHA for seven days prior to treatment with intranasal ODE or saline inhalations. Animals treated with 2 mg DHA demonstrated significant reductions in ODE-induced bronchial alveolar lavage neutrophil influx and pro-inflammatory cytokine/chemokine production compared to mice exposed to ODE alone. Collectively, these data demonstrate that DHA affects several lung cells to reduce the airway inflammatory response to organic dust exposures. Dietary supplementation with DHA may be an effective therapeutic strategy to reduce the airway inflammatory consequences in individuals exposed to agriculture dust environments.

## 1. Introduction

Exposure to complex organic dusts within agricultural environments leads to a variety of airway inflammatory diseases, including chronic bronchitis, organic dust toxic syndrome, rhinosinusitis, asthma, and chronic obstructive pulmonary disease [1,2,3,4]. Typical inflammatory reactions to acute organic dust exposure include increased inflammatory cytokine/chemokine responses and airway neutrophil influx in exposed workers [5]. Corticosteroid and β_2_ adrenergic receptor agonist therapies have been utilized in the treatment of inflammatory lung diseases resulting from agricultural dust exposures, but these therapies do not adequately prevent symptoms or disease progression [6]. Thus, a need exists to develop improved treatment options for preventing and/or halting lung inflammation and disease resulting from agricultural exposures.

Diets high in omega-3 fatty acids like docosahexaenoic acid (DHA) and eicosapentaenoic acid (EPA) are beneficial in a wide variety of conditions with underlying inflammatory components including cardiovascular disease and breast cancer [7,8]. As reviewed by Giudetti and Cagnazzo, omega-3 fatty acids have demonstrated benefits in several lung inflammatory conditions including asthma, acute respiratory distress syndrome, cystic fibrosis, and chronic obstructive pulmonary disease [9]. Recently, the mechanisms underlying the beneficial effects of these fatty acids have been at least partially explained with the discovery of omega-3 fatty acid-derived specialized pro-resolving mediators (SPM) [10,11,12,13]. SPMs, including resolvins, protectins and maresins, have specific anti-inflammatory and pro-resolving actions in a variety of cell types and tissues, including the ability to reduce inflammatory cytokine production, skew macrophage activation to an anti-inflammatory M2 phenotype, promote the non-phlogistic removal of neutrophils from sites of inflammation, and promote wound healing following tissue injury [14,15,16,17,18]. Within the lung, SPMs have exhibited beneficial effects in various preclinical models. These include the improvement of acute lung injury while maintaining host immunity, the reduction of neutrophilic influx and inflammatory cytokine production associated with cigarette smoke exposure, and the inhibition of asthmatic airway hyperresponsiveness [16,17,19]. Additionally, our group has shown that the SPM maresin-1 is effective at limiting the pro-inflammatory response of bronchial epithelial cells to organic dust exposures *in vitro* [20].

Based on these findings, we hypothesized that supplementation with the omega-3 fatty acid DHA may be a viable approach to reduce airway inflammation induced by agricultural organic dust exposures. To test this hypothesis, we assessed whether DHA pretreatment would reduce cytokine/chemokine responsiveness to organic dust extracts derived from swine confined animal feeding operations (ODE) utilizing human bronchial epithelial cells, monocytes, lung fibroblasts, and *ex vivo* precision-cut mouse lung slice cultures. Using our established animal inflammatory airway model [21], mice were also treated with DHA for one week prior to an intranasal inhalation challenge with ODE. Through these investigations, we found that supplementing the diets of mice with DHA for one week resulted in significant reductions in inflammatory mediator release caused by a single ODE instillation, while also decreasing ODE-induced airway neutrophilia. These data reveal the beneficial effects of the omega-3 fatty acid DHA in limiting airway inflammation associated with acute exposures to organic dusts and provide compelling support for future studies investigating their potential in ameliorating lung inflammation and disease associated with agricultural dust exposures.

## 2. Experimental Section

### 2.1. Reagents

Docosahexaenoic acid (DHA) and eicosapentaenoic acid (EPA) were obtained from Sigma-Aldrich (St. Louis, MO, USA). The human bronchial epithelial cell line, Beas-2B, human monocytic cell line, THP-1, and the human lung fibroblast cell line, HFL-1, were purchased from the American Type Culture Collection (Manassas, VA, USA). The anti-human PE-conjugated intracellular adhesion molecule-1 (ICAM-1) antibody was obtained from Biolegend (San Diego, CA, USA).

### 2.2. Swine Confinement Animal Feeding Operation Organic Dust Extract (ODE)

Settled dusts were obtained from swine confinement facilities, extracted into aqueous solvent, and characterized for inflammatory features, as previously described [22,23]. Briefly, dusts collected off surfaces at least one meter from the facility floor were suspended at 100 mg/mL in HBSS, centrifuged to remove insoluble particles and sterile filtered through a 0.22 μm filter. The resulting aqueous extract (100% ODE) was aliquoted and stored at −20 °C for future use.

### 2.3. Cell Culture

The human bronchial epithelial Beas-2B cell line was maintained in a 1:1 mixture of serum-free LHC basal medium (Life Technologies; Grand Island, NY, USA) supplemented with growth factors and RPMI medium (Sigma-Aldrich; St. Louis, MO, USA) as previously described [22]. Cells were plated on type I collagen-coated plates and utilized for experiments upon reaching ≥ 85% confluency. THP-1 monocytes and HFL-1 fibroblasts were maintained in DMEM supplemented with antibiotics and 10% fetal calf serum. Experiments were performed by adding 0–1 μM DHA to cell culture media for 1 hour prior to the addition of 1% ODE (THP-1) or 5% ODE (Beas-2B, HFL-1) to the DHA-treated media. Cell-free supernates for cytokine/chemokine level measurements were collected at 24 h (Beas-2B, THP-1) or 6 hours (HFL-1) post-stimulation with ODE.

### 2.4. Precision-Cut Mouse Lung Slice Cultures

Precision-cut mouse lung slice cultures were prepared as previously described [24]. Naïve C57BL/6 mice were euthanized and lungs were inflated with a low melting point agarose suspension. Lungs were transversely sectioned at a thickness of 150 μm using an Electron Microscopy Sciences OTS 4500 vibratome (Hatfield, PA, USA). Slices were cultured in LHC-9 growth medium at 37 °C, with replacement of growth medium to remove residual agarose and blood cells. After 4 to 6 days of equilibration, lung slice cultures were pretreated with 0–1 μM DHA for 18 h, and subsequently stimulated with 5% ODE for 6 h in the DHA-treated media. Supernates from lung slice cultures were collected for cytokine/chemokine measurements with normalization to homogenized lung slice protein levels.

### 2.5. Animal Care

Male C57BL/6 mice 6–8 weeks of age were obtained from The Jackson Laboratories (Bar Harbor, ME, USA) and housed in the University of Nebraska Medical Center (UNMC) Comparative Medicine Facilities under pathogen-free conditions. Mice were given unrestricted access to water and standard mouse chow. All experiments were approved and performed in accordance with the UNMC Institutional Animal Care and Use Committee.

### 2.6. Animal Model of ODE Exposure

For *in vivo* investigations, we utilized an established murine model of intranasal ODE inhalation-induced airway inflammation [21]. For these studies, mice were given 0 or 2 mg DHA in a mineral oil vehicle for seven consecutive days prior to a single 50 μL intranasal instillation of 12.5% ODE or saline solution. DHA dosing was chosen based on previously reported investigations of oral DHA dosing, as well as preliminary investigations for optimal dosing performed by our lab (data not shown). Previous reports have shown that incorporation of DHA into phospholipid membranes during dietary supplementation is similar between humans and mice [25]. Estimating a consumption of approximately 4 g of diet per day for adult C57Bl/6 mice [26], the 2 mg daily dose we used in this study is approximately equivalent to a human on a 2000 kcal diet with 30% calories from fat taking a 208 mg DHA supplement daily. At 5 h following ODE or saline treatment, mice were euthanized and bronchoalveolar lavage fluid (BALF) was obtained. Three fractions of 1 mL each were obtained, cell pellets were recovered, and the supernates from the first 1 mL of fluid were reserved for cytokine/mediator analyses. Cells from the three fractions were pooled and total cell numbers were enumerated following red blood cell lysis, and cells were cytocentrifuged with a Wescor Cytopro cytocentrifuge (Logan, UT, USA). Slides were stained using a Siemens Diff-Quik staining set (Newark, DE, USA) to determine inflammatory cell differentials.

### 2.7. Cytokine/Mediator Analyses

Cell-free supernates from cell cultures, mouse lung slice cultures, and mouse BALF were assayed for cytokine/mediator levels using enzyme-linked immunosorbant assays. Human cell line culture supernates were assayed for TNF-α, IL-6, and/or IL-8 using laboratory-designed immunoassays and murine cultures were assayed for murine TNF-α, IL-6, CXCL1 (KC), and CXCL2 (MIP-2) using assays and reagents obtained from R & D systems (*Duoset* EIA development kits, Minneapolis, MN, USA) and performed according to manufacturer’s directions. Levels of PGE_2_ and resolvin D1 were determined using enzyme immunoassay kits from Cayman Chemical (Ann Arbor, MI, USA) and run according to manufacturer’s directions. Limits of detectability were as follows: 125 pg/mL for IL-8 (human), 35–60 pg/mL for IL-6 (human), 15 pg/mL for TNF-α (human), 35 pg/mL for IL-6 (murine), 25 pg/mL for TNF-α (murine), 15 pg/mL for CXCL1 (murine), 52–60 pg/mL for CXCL2 (murine), 15 pg/mL for PGE_2_, and 15 pg/mL for RvD1.

### 2.8. Flow Cytometry

Beas-2B cells were pretreated with 0–1 μM DHA for 1 h prior to stimulation with 5% ODE for 24 h. Cells were gently detached from plates with a warmed EDTA solution, washed in PBS, and fixed with 1% paraformaldehyde. Cells were then stained for 1 h with PE-conjugated anti-human ICAM-1 antibodies (BD Biosciences, San Jose, CA, USA) or isotype control antibody as previously described [27]. Cell surface expression of ICAM-1 was determined after excluding debris by antibody-specific mean fluorescence intensity (MFI) of 10,000 gated events per group using a BD FACSCalibur dual laser cytometer, run by the UNMC Cell Analysis Core Facility in Omaha, NE, USA). Analyses were performed using the BD CellQuest software (BD Biosciences, San Jose, CA, USA) and De Novo flow cytometry analysis software (Glendale, CA, USA).

### 2.9. Statistical Analyses

Prism Software by Graphpad (San Diego, CA, USA) was utilized for graphing and analyses. Data are reported as mean +/− standard error of the mean. Statistical significance was determined using one-way ANOVA with Tukey’s post hoc analyses and significance was established as *p* ≤ 0.05. Cell and tissue culture studies were performed in duplicate replicates with a minimum of three independent runs. *In vivo* experiments included 10 mice per group for cell counts/cytokine analyses, and three (saline controls) to four (ODE/DHA treatment groups) BALF samples from each group assayed twice for lipid mediator analyses.

## 3. Results

### 3.1. DHA Pretreatment Reduces Organic Dust-Induced Inflammatory Cytokine/Chemokine Production from Bronchial Epithelial Cells, Monocytes, and Fibroblasts

**Figure 1:**
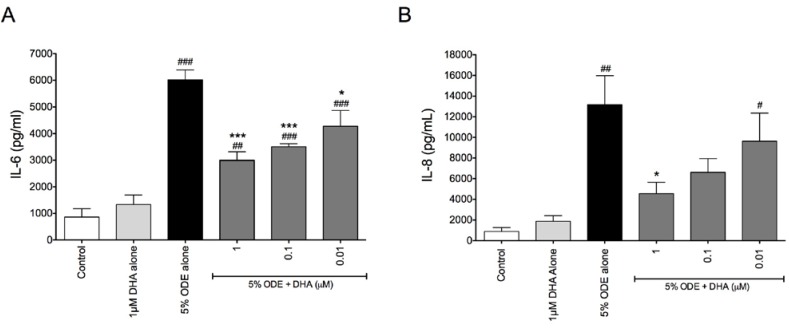
DHA pretreatment reduces ODE-stimulated IL-6 and IL-8 release in bronchial epithelial cells. Beas-2B cells were pretreated for 1 h with 0, 0.01, 0.1, or 1 μM DHA prior to stimulation with 5% ODE. At 24 h following ODE treatment, supernates were collected and IL-6 (**A**) and IL-8 (**B**) release were measured via ELISA. ^#^ *p* ≤ 0.05 *vs.* control; ^##^ *p* ≤ 0.01 *vs.* control; ^###^ *p* ≤ 0.001 *vs.* control; * *p* ≤ 0.05 *vs.* 5% ODE alone; *** *p* ≤ 0.001 *vs.* 5% ODE alone.

We have previously reported that exposure of bronchial epithelial cells and monocytes to ODE lead to pro-inflammatory responses, including the production of pro-inflammatory cytokines and chemokines [22,28]. In these investigations, we assessed how DHA pre-treatment of bronchial epithelial cells, monocytes, and lung fibroblasts prior to ODE exposure would alter the responses of these cell types to the dust. When Beas-2B human bronchial epithelial cells were treated for 1 h with 0–1 μM DHA prior to stimulation with 5% ODE for 24 h, DHA-pretreated cells had significantly reduced ODE-induced IL-6 release compared to cells treated with ODE alone (Figure 1A). Cells receiving 1 μM DHA pretreatment also exhibited significantly reduced IL-8 release compared to cells treated with 5% ODE only (Figure 1B).

**Figure 2:**
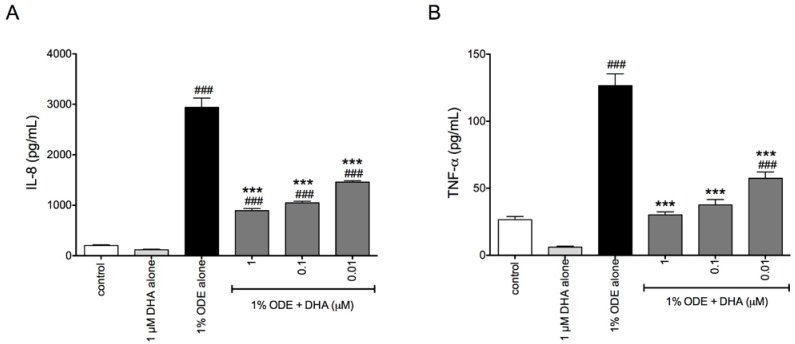
DHA pretreatment reduces ODE-stimulated IL-8 and TNF-α release in monocytes. THP-1 cells were pretreated for 1 h with 0, 0.01, 0.1, or 1 μM DHA prior to stimulation with 1% ODE. At 24 h after ODE treatment, cell supernates were collected and IL-8 (**A**) and TNF-α release (**B**) were measured via ELISA. ^###^ *p* ≤ 0.001 *vs.* control; *** *p* ≤ 0.001 *vs.* 5% ODE alone.

In addition to the inflammatory response seen in bronchial epithelial cells to organic dusts, previous data have shown that monocytes significantly increase their release of IL-8 and TNF-α in response to ODE [28]. When we pretreated THP-1 monocytes for 1 h with 0–1 μM DHA followed by 24 h treatment with 1% ODE, DHA-treated cells exhibited significant dose-dependent reductions in ODE-stimulated production of both IL-8 and TNF-α compared to cells receiving ODE treatment alone (Figure 2).

**Figure 3:**
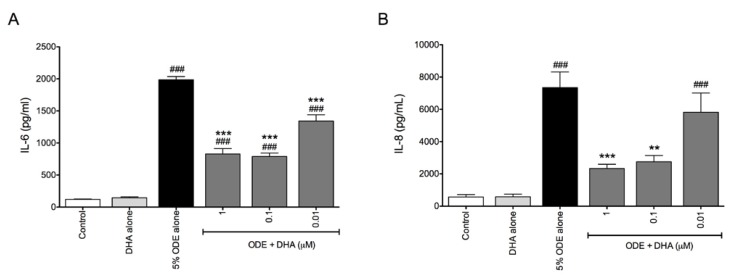
DHA pretreatment reduces ODE-stimulated IL-6 and IL-8 release in lung fibroblasts. HFL-1 cells were pretreated for 18 h with 0, 0.01, 0.1, or 1 μM DHA prior to treatment with 5% ODE. At 6 hours following ODE stimulation, supernates were collected and IL-6 (**A**) and IL-8 (**B**) release were measured via ELISA. ^###^ *p* ≤ 0.001 *vs.* control; ** *p* ≤ 0.01 *vs.* 5% ODE alone; *** *p* ≤ 0.001 *vs.* 5% ODE alone.

During inflammatory processes, lung fibroblasts play important roles in both regulating immune response through the secretion of cytokines and upregulation of adhesion molecules for leukocyte interactions, as well as contributing to wound healing and tissue integrity through the release of matrix metalloproteinases as well as extracellular matrix constituents [29,30]. To further analyze the effect of ODE and DHA supplementation on lung cell types, we utilized the lung fibroblast HFL-1 cell line. When HFL-1 cells were stimulated with 5% ODE for 6 h, their release of IL-6 and IL-8 was significantly increased compared to untreated cells (Figure 3). However, DHA-pretreated cells had significantly reduced IL-6 and IL-8 release compared to those cells stimulated with ODE alone (Figure 3). To extend our findings to another omega-3 fatty acid, we also tested the effect of EPA treatment on ODE-stimulated cytokine production in HFL-1 cells. Similarly to DHA, EPA was also effective at significantly reducing ODE-stimulated IL-6 and IL-8 release in HFL-1 fibroblasts (data not shown). Together, these data indicate a role for the omega-3 fatty acid DHA in reducing ODE-induced cytokine/chemokine release in multiple cell types relevant to lung cellular responses to airway injury signals.

### 3.2. DHA Pretreatment Reduces ODE-Induced Inflammatory Cytokine Production in Ex Vivo Precision-Cut Mouse Lung Slice Cultures

**Figure 4:**
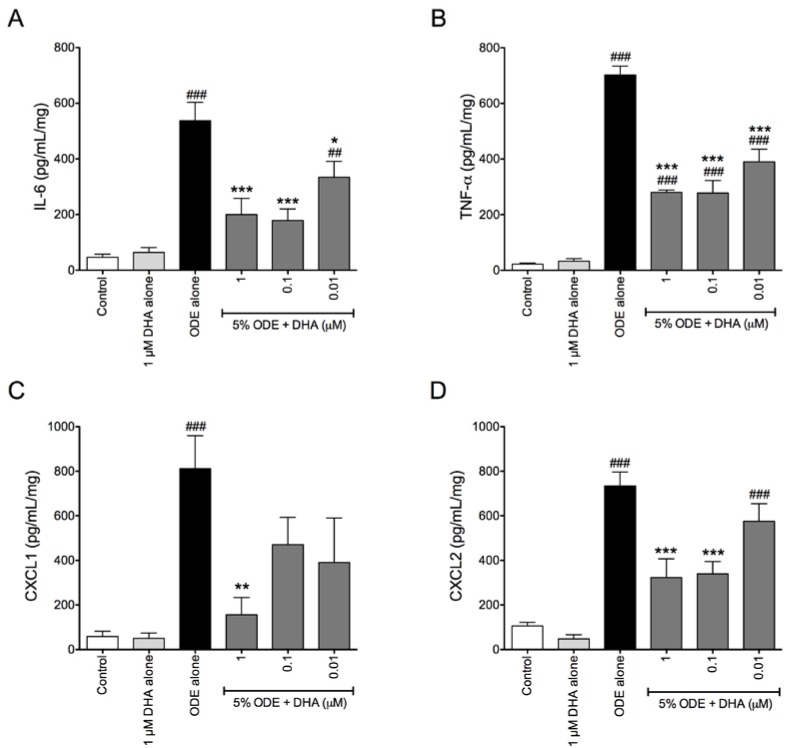
DHA pretreatment reduces ODE-induced IL-6, TNF-α, CXCL1, and CXCL2 release from precision-cut mouse lung slices. Precision-cut mouse lung slices were pretreated for 18 h with 0, 0.01, 0.1, or 1 μM DHA prior to 6 h treatment with 5% ODE. Following 6 h treatment, supernates were collected from the lung slices and IL-6 (**A**); TNF-α (**B**); CXCL1 (**C**); and CXCL2 (**D**) levels were determined via ELISA. ^##^ *p* ≤ 0.01 *vs.* control; ^###^ *p* ≤ 0.001 *vs.* control; * *p* ≤ 0.05 *vs.* 5% ODE alone; ** *p* ≤ 0.01 *vs.* 5% ODE alone; *** *p* ≤ 0.001 *vs.* 5% ODE alone.

To extend our *in vitro* investigations, we examined the effects of DHA treatment prior to ODE exposure in an *ex vivo* precision-cut mouse lung slice model. This model system allows for investigation of lung inflammatory responses while lung cells, extracellular matrix, and other lung tissue components remain in contact with each other. This facilitates a more realistic system with which to assess the total lung response to an inflammatory stimulus as compared to traditional cell culturing methods. We have previously reported that treatment with 5% ODE stimulates significant production of TNF-α, IL-6, and CXCL1 in the mouse lung slice model [20]. However, when lung slice cultures were pretreated with 0.1 and 1 μM DHA for 18 h prior to 5% ODE stimulation, TNF-α, IL-6, CXCL1, and CXCL2 inflammatory mediator production was significantly reduced at 6 h following ODE exposure, as compared to ODE treatment alone (Figure 4). These data support a role for DHA in an *ex vivo* model system whereby DHA treatment prior to dust exposure significantly reduced the inflammatory response of the lung slices to the ODE stimulus.

### 3.3. DHA Pretreatment Decreases ODE-Induced ICAM-1 Up-Regulation on Bronchial Epithelial Cells

**Figure 5:**
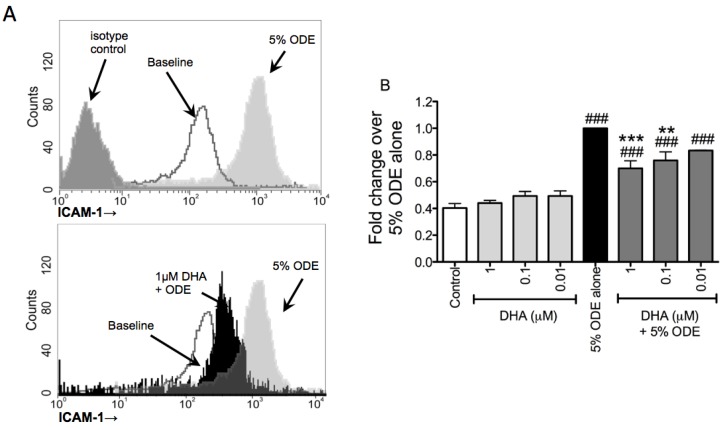
DHA pretreatment reduces ODE-induced ICAM-1 expression on bronchial epithelial cells. Beas-2B cells were pretreated with 0. 0.01, 0.1, or 1 μM DHA for 1 h prior to stimulation with 5% ODE for 24 h. Following 24 h ODE treatment, cells were stained for ICAM-1 expression and flow cytometry was performed to delineate the ICAM-1 surface expression levels on the bronchial epithelial cells. (**A**) Gated histogram of representative experiment indicating the intensity of PE staining on bronchial epithelial cells with and without DHA and ODE treatments; (**B**) Bar graph summarizing three separate experiments indicating the fold change of mean fluorescent intensities over 5% ODE alone. Data were calculated from 10,000 gated events for each treatment group within each experiment following normalization with isotype controls. ^###^ *p* ≤ 0.001 *vs.* control; ** *p* ≤ 0.01 *vs.* 5% ODE alone; *** *p* ≤ 0.001 *vs.* 5% ODE alone.

The epithelial cell adhesion molecule ICAM-1 facilitates neutrophil adherence and interaction with the bronchial epithelium that is important for the inflammatory response of these cells in the lungs [31,32]. We have previously found that treatment of bronchial epithelial cells with ODE increased the expression of ICAM-1 on the cells and correlates with greater lymphocyte [27] and neutrophil (unpublished observation) adhesion to the epithelial cells. When Beas-2B cells were pretreated with 0.1 or 1 μM DHA prior to stimulation with 5% ODE for 24 h, the upregulation of ICAM-1 (mean fluorescence intensity; MFI) on the surface of the bronchial epithelial cells was blunted in the DHA-pretreated cells as compared to the cells receiving only ODE treatment (Figure 5). These data indicate DHA is modulating the pro-inflammatory response of bronchial epithelial cells not only at the level of cytokine production, but by limiting their ODE-induced adhesion molecule expression as well.

### 3.4. One-Week Supplementation with DHA Reduces the Airway Inflammatory Response of Mice to ODE Inhalation

**Figure 6:**
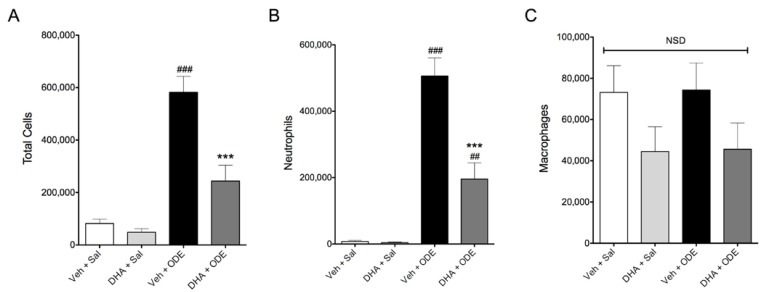
Effect of DHA supplementation on cell influx into the airways of mice receiving a single intranasal ODE instillation. C57BL/6 mice were given daily 0 or 2 mg DHA supplementation via oral gavage for seven consecutive days prior to a single intranasal instillation of 50 μL 12.5% ODE or saline and BALF was obtained 5 h post-treatment. Total (**A**); neutrophil (**B**); and macrophage (**C**) cell numbers were enumerated. ^##^ *p* ≤ 0.01 *vs.* vehicle + saline; ^###^ *p* ≤ 0.001 *vs.* vehicle + saline; *** *p* ≤ 0.001 *vs.* vehicle + ODE. NSD: no statistical difference. *N* = 10 mice per group.

**Figure 7:**
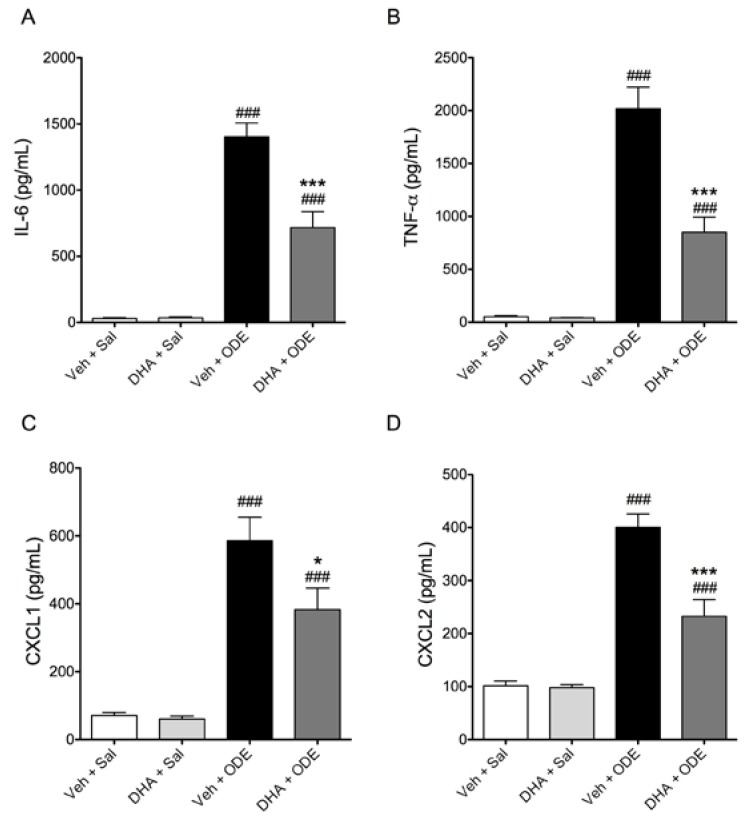
DHA supplementation reduces inflammatory cytokine and chemokine release in mice receiving a single intranasal ODE instillation. C57BL/6 mice were given daily 0 or 2 mg DHA supplementation via oral gavage for seven consecutive days prior to a single intranasal instillation of 50 μL 12.5% ODE or saline. At 5 h following ODE treatment, BALF was obtained and assayed for IL-6 (**A**); TNF-α (**B**); CXCL1 (**C**); and CXCL2 (**D**) via ELISA. ^###^ *p* ≤ 0.001 *vs.* vehicle + saline; * *p* ≤ 0.05 *vs.* vehicle + ODE; *** *p* ≤ 0.001 *vs.* vehicle + ODE. *N* = 10 mice per group.

**Figure 8:**
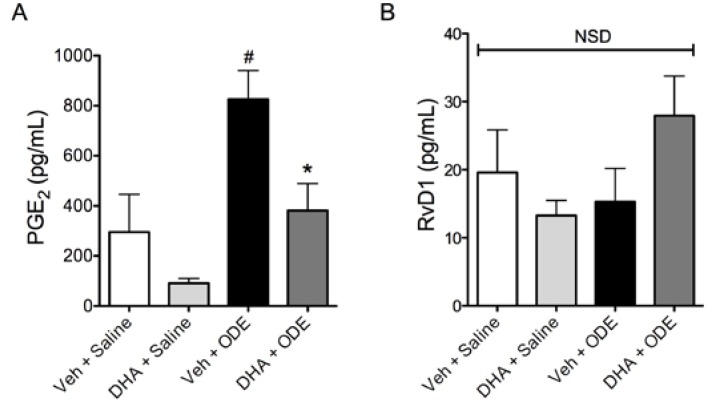
Effect of DHA supplementation on bioactive lipid mediator production in mice receiving a single intranasal ODE instillation. C57BL/6 mice were given 0 or 2 mg orally for seven consecutive days prior to a single intranasal challenge of 50 μL 12.5% ODE or saline. Five hours after ODE exposure, BALF was obtained and enzyme immunoassays were used to detect PGE_2_ (**A**) and RvD1 (**B**) levels in the lavage fluids. ^#^ *p* ≤ 0.05 *vs.* vehicle + saline; * *p* ≤ 0.05 *vs.* vehicle + ODE. *N* = 3 mice in Veh + Saline and DHA + Saline groups; *N* = 4 mice in Veh + ODE and DHA + ODE groups.

Acute exposures to swine confinement facility dusts lead to airway inflammation as evidenced by increased inflammatory mediator levels and neutrophilia in the bronchoalveolar lavage fluid (BALF) of exposed individuals [5]. To investigate how acute organic dust exposures cause airway inflammation, we have previously developed and reported a murine model of acute ODE exposure [21], where a single intranasal instillation of ODE leads to inflammatory cytokine release and neutrophil influx in the BALF of ODE-instilled mice. When we gave C57BL/6 mice daily doses of 0 or 2 mg DHA via oral gavage for seven consecutive days prior to a single 50 μl intranasal instillation of 12.5% ODE or saline, we found that DHA-treated mice had reduced total BALF cell numbers and neutrophil influx induced by ODE treatment (Figure 6). Furthermore, mice treated with DHA had significant reductions in BALF IL-6, TNF-α, CXCL1, and CXCL2 levels following ODE treatment as compared to vehicle-supplemented mice (Figure 7). In addition to inflammatory cytokine and chemokine release, we also assayed for the omega-6 fatty acid-derived lipid mediator PGE_2_ in BALF and found that while ODE exposure stimulated PGE_2_ production, DHA supplementation significantly reduced the BALF levels of this lipid mediator (Figure 8A). Because pro-inflammatory mediator production was significantly reduced in DHA-pretreated mice, including the omega-6 fatty acid-derived PGE_2_, we sought to determine if DHA supplementation would modify BALF levels of a lipid mediator derived from DHA. Specifically, we assessed whether the DHA-derived pro-resolving lipid mediator resolvin D1 (RvD1) levels would be altered in DHA-supplemented mice with or without exposure to ODE. Somewhat surprisingly, neither a single exposure to ODE or 1 week of daily supplementation with DHA had a significant effect on BALF RvD1 levels (Figure 8B). Collectively, these data indicate that DHA modulates lung inflammation by reducing inflammatory mediator production and decreasing neutrophil influx into the airways of ODE-instilled mice.

## 4. Discussion

Exposure to the complex dusts found in agricultural environments leads to airway inflammatory diseases [1,2]. In recent years, omega-3 fatty acids were found to play an important role in the endogenous circuits regulating the resolution of inflammation, wherein omega-3 fatty acid-derived bioactive lipid mediators orchestrate anti-inflammatory and pro-resolving processes in a variety of inflammatory settings, including lung inflammation [9,12,13]. We therefore posited that supplementation with omega-3 fatty acids might facilitate enhanced anti-inflammatory and pro-resolving pathways in the lung during agricultural dust-induced airway inflammation that could mitigate the negative consequences of these inflammatory exposures. Through these investigations, we have found that DHA treatment leads to decreased organic dust-induced airway inflammation through actions on various lung cell types. These data provide compelling support for omega-3 supplementation as a strategy for reducing inflammation associated with agricultural dust exposures.

Within the lung, the first contact that occurs with exogenous irritants often occurs at the level of the bronchial epithelial cell. Acting as more than just a barrier, bronchial epithelial cells are activated by exogenous stimulants to orchestrate an inflammatory response against the foreign substances to which they are exposed [33,34]. We have previously shown that organic dust treatment of bronchial epithelial cells activates protein kinase C (PKC)-α and PKC-ε and causes the transcriptional activation of NFκB and MAPK transcription factors [20,35]. ICAM-1 expression is upregulated, along with increased release of inflammatory cytokines and chemokines, including IL-6 and IL-8 [22,27]. These bronchial epithelial cell changes are important to the development of airway inflammation, as the inflammatory cytokines serve to recruit and activate neutrophils and macrophages in the lung while ICAM-1 expression on the airway epithelial cells facilitates interactions with the incoming leukocytes [21,36,37]. Together, these bronchial epithelial cell changes encourage the airway inflammatory response that is seen with exposures to organic dusts. Importantly, SPM derived from omega-3 fatty acids have been shown to modulate inflammation via interactions at several of these steps. At the level of the airway epithelium, we have shown the DHA-derived SPM maresin-1 reduces PKC-α and PKC-ε activation and subsequent IL-6 and IL-8 release from bronchial epithelial cells [20]. Gong and colleagues found decreases in inflammatory cytokine release, epithelial cell ICAM-1 expression, and airway neutrophilia with maresin-1 in the context of LPS-induced acute lung injury [38]. Another DHA-derived SPM, RvD1, also modulates various aspects of airway inflammation, including reducing the production of pro-inflammatory cytokines, polarizing macrophages to an anti-inflammatory M2 phenotype, and reducing the influx and promoting the clearance of neutrophils from the lungs following airway injury [16,39].

Consistent with these prior studies of DHA-derived SPM, we found that DHA treatment reduced IL-6 and IL-8 release induced by ODE challenge in bronchial epithelial cells. Furthermore, the ODE-induced upregulation of bronchial epithelial cell ICAM-1 expression was also diminished with DHA treatment. We also demonstrated that the ability of DHA to reduce ODE-induced cytokine/chemokine production was not limited to bronchial epithelial cells, but extends to mononuclear phagocytes, fibroblasts, and whole lung tissues as well. These data together indicate a protective role for DHA treatment at the cellular and lung tissue levels for the prevention of organic dust-induced airway inflammation.

Based on these findings, we hypothesized that dietary supplementation with DHA *in vivo* would lead to reduced airway inflammation from an organic dust exposure. There are several human studies demonstrating that dietary supplementation with omega-3 fatty acids is beneficial in preventing or limiting airway inflammatory disease [9]. A meta-analysis review of clinical trial reports found that omega-3 fatty acid supplementation reduces mortality rates amongst other physiological outcomes in patients suffering from acute lung injury and acute respiratory distress syndrome [40]. Additionally, omega-3 fatty acid supplementation increased lung function, reduced sputum levels, and lowered leukotriene B4 to B5 ratios in supplemented patients with cystic fibrosis [41,42]. Thus, to investigate the role of DHA supplementation in modulating organic dust-induced airway inflammation *in vivo*, we supplemented the standard rodent chow diet of mice for 1 week by orally administering 2 mg of DHA prior to being challenged with an intranasal instillation of 12.5% ODE. As compared to mice receiving only vehicle supplementation (mineral oil) and ODE exposure, mice that received DHA supplementation showed marked reductions in the total inflammatory cell infiltration into the airways, corresponding to reduced neutrophil influx following the ODE exposure. This correlated with the reduced release of neutrophil chemoattractants CXCL1 and CXCL2 in the bronchoalveolar lavage fluid, as well as reduced pro-inflammatory IL-6 and TNF-α cytokines in DHA + ODE treated mice as compared to vehicle + ODE treated mice. Together, these data provide compelling evidence for a beneficial effect of DHA dietary supplementation in mitigating acute organic dust-induced airway inflammation. A limitation of these studies is that the effect of DHA supplementation on chronic airway inflammation, such as that which would be experienced by agriculture workers, was not investigated. Mouse models of chronic lung inflammatory conditions reveal variable results regarding the use of DHA alone in reducing lung inflammation. In a mouse model of bleomycin-induced pulmonary fibrosis, mice receiving intra-tracheal DHA had reduced lung inflammation and injury [43]. However, in a mouse model of allergic asthma, mice on a high DHA diet had indicators of increased inflammation over those on control or EPA +/− DHA diets [44]. Therefore, it will be important to test the efficacy of DHA supplementation in a model of organic dust-induced chronic airway inflammation to elucidate the fatty acid’s potential in preventing and treating dust-induced lung inflammatory diseases.

DHA and other omega-3 fatty acids are recognized to propagate their cellular effects through direct binding and activation of the omega-3 fatty acid receptor GPR120 on cell surfaces, as well as through incorporation and subsequent release of the fatty acid from the plasma membranes of cells following stimulation [45,46,47,48,49]. In the former scenario, GPR120 activation inhibits the phosphorylation and activation of the MAP3 kinase TAK1 [45,50]. Interestingly, exposure to ODE activates pro-inflammatory signaling via activation of TLR2 and TNFR, amongst other cell surface receptors, leading to the activation of NFκB and MAPK transcriptional pathways [20,35,51,52]. A key converging point in these signaling pathways is the activation of the MAP3 kinase TAK1 [53,54,55]. One potential mechanism of DHA anti-inflammatory action may be to abrogate NFκB and MAPK signaling via blocking TAK1 activation. Future studies to investigate this potential mechanism are warranted.

As previously mentioned, DHA and other omega-3 fatty acids can also alter cell functioning through incorporation and subsequent release from the cell membrane following stimulation [47,48]. In this scenario, supplementation with DHA leads to an increased ratio of DHA to arachidonic acid (omega-6 fatty acid) in the plasma membrane of cells [46,49]. When cells become activated, *i.e.*, by a pro-inflammatory stimulus, the cPLA_2_ enzyme will liberate fatty acids from the cell membrane for use in the production of bioactive lipid mediators. In the case of arachidonic acid, many major metabolites are pro-inflammatory mediators including prostaglandins and cysteinyl leukotrienes, while omega-3 fatty acids like DHA are metabolized into pro-resolving mediators including resolvins and maresins [56,57]. Thus, increasing the proportion of DHA to arachidonic acid in the plasma membrane could shift the bioactive lipid mediator balance from pro-inflammatory to pro-resolving during an inflammatory response.

With this mechanism of DHA action in mind, we analyzed the levels of the arachidonic acid lipid metabolite, PGE_2_, as well as a DHA lipid metabolite, RvD1, in the lavage fluids of the mice following ODE exposure. Mice that received no DHA supplementation prior to a single ODE instillation had significant increases in PGE_2_ in their BALF compared to saline-instilled mice, and this response was completely abolished in the DHA supplemented animals. However, no significant differences were detectable in DHA-derived RvD1 levels in the BALF of mice across treatment groups. There appeared to be a trend, however, towards increased RvD1 levels in DHA supplemented mice receiving ODE compared to mice receiving no DHA prior to ODE exposure. It remains a possibility that our time frame for analyzing RvD1 levels in BALF (5 h post ODE exposure) was not appropriate for capturing the production of this mediator, or that an enhanced production and immediate utilization of RvD1 within the *in vivo* system resulted in low apparent levels of the mediator in the lavage. In addition, we did not measure additional DHA-derived SPM, including MaR1, which has been previously shown to reduce organic dust-induced inflammatory signaling in BECs [20]. Thus, it remains a possibility that while RvD1 levels were not significantly altered with DHA supplementation, other SPM may be responsible for the anti-inflammatory effects seen in our *in vivo* investigations. Based on our current findings however, it is unclear if one week of DHA supplementation is modifying the polyunsaturated fatty acid composition of cell membranes sufficiently to alter bioactive lipid mediator production during an inflammatory response. It remains a clear possibility that the anti-inflammatory effects of DHA supplementation that have been found are due to other mechanisms of DHA action within the lung milieu. Future studies are warranted to further investigate the mechanisms underlying the actions of DHA supplementation in the lung that can better define their protective actions against organic dust-induced airway inflammation.

## 5. Conclusions

In conclusion, these investigations identify a role for DHA supplementation in mitigating the lung inflammatory effects of organic dust exposure. The ease of incorporating DHA into one’s diet either by supplementation or diet change make these findings particularly valuable in considering novel options for preventing or treating airway inflammation and disease associated with agricultural exposures. Future studies investigating how longer-term DHA supplementation and/or supplementation during repetitive organic dust exposures affect airway inflammatory outcomes will shed light on the potential of this dietary fatty acid in improving or preventing lung inflammation and disease for individuals exposed to agricultural dusts.
